# Supplementary material for: Comparative multiomics analysis of cell physiological state after culture in a basket bioreactor
Source: Sci Rep. 2022 Nov 23;12:20161. doi: 10.1038/s41598-022-24687-4 (PMC9686226; doi:10.1038/s41598-022-24687-4)
Supplement: Supplementary file 1 — Supplementary Information 1. [file 41598_2022_24687_MOESM1_ESM.zip › raw data/Proteomic raw data/6.KEGGenrichment/EG.vs.CG/DOWN/pathway/KEGG.map.html]

Pathway Enrichment


  

# The most enriched pathway terms of EG.vs.CG

Statistic method: hypergeometric test

FDR correction method: Benjamini and Hochberg

| MapID | MapTitle | Pvalue | AdjustedPv | x | y | n | N | EnrichDirect | ProtID | Description  | |
| --- | --- | --- | --- | --- | --- | --- | --- | --- | --- | --- | --- |
| map04514 | Cell adhesion molecules (CAMs) | 6.10780043080542e-07 | 6.65750246957791e-05 | 9 | 44 | 69 | 2848 | Over | A0A0D9R3G8 A0A0D9R457 A0A0D9R6A4 A0A0D9RNI2 A0A0D9RQI3 A0A0D9RYA9 A0A0D9S2Z2 A0A0D9S368 A0A0D9S6M6 | Uncharacterized protein,Uncharacterized protein,Uncharacterized protein,Ig-like domain-containing protein,B-cell surface antigen CD40,Protein-tyrosine-phosphatase,Neural cell adhesion molecule 1,Uncharacterized protein,Uncharacterized protein |
| map04610 | Complement and coagulation cascades | 0.000747521430993758 | 0.0407399179891598 | 4 | 18 | 69 | 2848 | Over | A0A0D9QZZ6 A0A0D9REV5 A0A0D9RRB2 A0A0D9RTW6 | VWFA domain-containing protein,SERPIN domain-containing protein,Plasminogen activator,Clusterin |
| map04512 | ECM-receptor interaction | 0.00133154213811844 | 0.0483793643516365 | 5 | 35 | 69 | 2848 | Over | A0A0D9R486 A0A0D9R9X4 A0A0D9RY11 A0A0D9RY12 A0A0D9S8B1 | Collagen type IV alpha 3 chain,Fibronectin,Collagen type IV alpha 2 chain,Collagen IV NC1 domain-containing protein,Uncharacterized protein |
| map04974 | Protein digestion and absorption | 0.00232595259623791 | 0.0633822082474831 | 4 | 24 | 69 | 2848 | Over | A0A0D9R486 A0A0D9RY11 A0A0D9RY12 A0A0D9S6M1 | Collagen type IV alpha 3 chain,Collagen type IV alpha 2 chain,Collagen IV NC1 domain-containing protein,Fibrillar collagen NC1 domain-containing protein |
| map00600 | Sphingolipid metabolism | 0.00363692003932437 | 0.0683594114830611 | 4 | 27 | 69 | 2848 | Over | A0A0D9QVQ6 A0A0D9QWC2 A0A0D9RVZ2 A0A0D9S760 | Sphingomyelin phosphodiesterase,Sphingomyelin phosphodiesterase,Ceramidase,Phospholipid phosphatase 3 |
| map05146 | Amoebiasis | 0.00376290338438868 | 0.0683594114830611 | 5 | 44 | 69 | 2848 | Over | A0A0D9R486 A0A0D9R9X4 A0A0D9RQT0 A0A0D9RY11 A0A0D9RY12 | Collagen type IV alpha 3 chain,Fibronectin,Uncharacterized protein,Collagen type IV alpha 2 chain,Collagen IV NC1 domain-containing protein |
| map04933 | AGE-RAGE signaling pathway in diabetic complications | 0.00550530732555996 | 0.0857254997837193 | 5 | 48 | 69 | 2848 | Over | A0A0D9R486 A0A0D9R9X4 A0A0D9RY11 A0A0D9RY12 A0A0D9S6M6 | Collagen type IV alpha 3 chain,Fibronectin,Collagen type IV alpha 2 chain,Collagen IV NC1 domain-containing protein,Uncharacterized protein |
| map00910 | Nitrogen metabolism | 0.00814917349632405 | 0.0986955456777023 | 2 | 6 | 69 | 2848 | Over | A0A0D9RIT7 A0A0D9RQX6 | Carbonic anhydrase,Carbonic anhydrase |
| map04672 | Intestinal immune network for IgA production | 0.00814917349632405 | 0.0986955456777023 | 2 | 6 | 69 | 2848 | Over | A0A0D9R6A4 A0A0D9RQI3 | Uncharacterized protein,B-cell surface antigen CD40 |
| map00500 | Starch and sucrose metabolism | 0.00998820752550342 | 0.108871462027987 | 3 | 19 | 69 | 2848 | Over | A0A0D9R6H1 A0A0D9RT09 A0A0D9S336 | Glycogen [starch] synthase,Uncharacterized protein,Glycogen [starch] synthase |
| map04060 | Cytokine-cytokine receptor interaction | 0.013243738317697 | 0.131233406966271 | 3 | 21 | 69 | 2848 | Over | A0A0D9RQI3 A0A0D9S6I0 A0A0D9S8Z3 | B-cell surface antigen CD40,Macrophage colony-stimulating factor 1,TNF receptor superfamily member 18 |
| map05222 | Small cell lung cancer | 0.0222791888399742 | 0.202369298629766 | 4 | 45 | 69 | 2848 | Over | A0A0D9R486 A0A0D9R9X4 A0A0D9RY11 A0A0D9RY12 | Collagen type IV alpha 3 chain,Fibronectin,Collagen type IV alpha 2 chain,Collagen IV NC1 domain-containing protein |
| map05310 | Asthma | 0.0242275280898877 | 0.203138504753673 | 1 | 1 | 69 | 2848 | Over | A0A0D9RQI3 | B-cell surface antigen CD40 |
| map04151 | PI3K-Akt signaling pathway | 0.0321206089844758 | 0.250081884236276 | 7 | 127 | 69 | 2848 | Over | A0A0D9R486 A0A0D9R6H1 A0A0D9R9X4 A0A0D9RY11 A0A0D9RY12 A0A0D9S336 A0A0D9S6I0 | Collagen type IV alpha 3 chain,Glycogen [starch] synthase,Fibronectin,Collagen type IV alpha 2 chain,Collagen IV NC1 domain-containing protein,Glycogen [starch] synthase,Macrophage colony-stimulating factor 1 |
| map04064 | NF-kappa B signaling pathway | 0.0442249895846601 | 0.300202668381482 | 3 | 33 | 69 | 2848 | Over | A0A0D9RQI3 A0A0D9RWK4 A0A0D9S6M6 | B-cell surface antigen CD40,Ubiquitinyl hydrolase 1,Uncharacterized protein |
| map04668 | TNF signaling pathway | 0.0442249895846601 | 0.300202668381482 | 3 | 33 | 69 | 2848 | Over | A0A0D9RWK4 A0A0D9S6I0 A0A0D9S6M6 | Ubiquitinyl hydrolase 1,Macrophage colony-stimulating factor 1,Uncharacterized protein |
| map00830 | Retinol metabolism | 0.0495747525767586 | 0.300202668381482 | 2 | 15 | 69 | 2848 | Over | A0A0D9RMB0 A0A0D9S8K6 | NADP-retinol dehydrogenase,Uncharacterized protein |
| map05144 | Malaria | 0.0495747525767586 | 0.300202668381482 | 2 | 15 | 69 | 2848 | Over | A0A0D9RQI3 A0A0D9S6M6 | B-cell surface antigen CD40,Uncharacterized protein |
| map04978 | Mineral absorption | 0.0830624179504208 | 0.463211708141373 | 2 | 20 | 69 | 2848 | Over | A0A0D9RJ61 A0A0D9S334 | Ferric oxidoreductase domain-containing protein,Ferritin |
| map04360 | Axon guidance | 0.0877241919318047 | 0.463211708141373 | 4 | 70 | 69 | 2848 | Over | A0A0D9R3G8 A0A0D9R4M5 A0A0D9R710 A0A0D9RQF9 | Uncharacterized protein,Uncharacterized protein,Plexin A1,Protein-serine/threonine kinase |
| map04621 | NOD-like receptor signaling pathway | 0.0913321624138765 | 0.463211708141373 | 4 | 71 | 69 | 2848 | Over | A0A0D9QWT4 A0A0D9RR35 A0A0D9RWK4 A0A0D9S6T5 | Anthrax toxin receptor,VWFA domain-containing protein,Ubiquitinyl hydrolase 1,GB1/RHD3-type G domain-containing protein |
| map05340 | Primary immunodeficiency | 0.0934922713679836 | 0.463211708141373 | 1 | 4 | 69 | 2848 | Over | A0A0D9RQI3 | B-cell surface antigen CD40 |
| map04926 | Relaxin signaling pathway | 0.124624231566696 | 0.588943696473012 | 3 | 51 | 69 | 2848 | Over | A0A0D9R486 A0A0D9RY11 A0A0D9RY12 | Collagen type IV alpha 3 chain,Collagen type IV alpha 2 chain,Collagen IV NC1 domain-containing protein |
| map04657 | IL-17 signaling pathway | 0.129675676287636 | 0.588943696473012 | 2 | 26 | 69 | 2848 | Over | A0A0D9RNH8 A0A0D9RWK4 | Lipocalin 2,Ubiquitinyl hydrolase 1 |
| map04931 | Insulin resistance | 0.135597782367563 | 0.591206331122574 | 3 | 53 | 69 | 2848 | Over | A0A0D9R6H1 A0A0D9RU34 A0A0D9S336 | Glycogen [starch] synthase,CBM21 domain-containing protein,Glycogen [starch] synthase |
| map04142 | Lysosome | 0.153384369927101 | 0.614707563837828 | 4 | 86 | 69 | 2848 | Over | A0A0D9QWC2 A0A0D9RBZ8 A0A0D9RVZ2 A0A0D9S6J4 | Sphingomyelin phosphodiesterase,Cathepsin D,Ceramidase,Sortilin 1 |
| map00730 | Thiamine metabolism | 0.157906530160176 | 0.614707563837828 | 1 | 7 | 69 | 2848 | Over | A0A0D9S8B5 | Alkaline phosphatase |
| map04080 | Neuroactive ligand-receptor interaction | 0.157906530160176 | 0.614707563837828 | 1 | 7 | 69 | 2848 | Over | A0A0D9RJD8 | KISS1 receptor |
| map00920 | Sulfur metabolism | 0.198321055166142 | 0.7057128651632 | 1 | 9 | 69 | 2848 | Over | A0A0D9S1A5 | Lactamase\_B domain-containing protein |
| map05152 | Tuberculosis | 0.201114567142638 | 0.7057128651632 | 3 | 64 | 69 | 2848 | Over | A0A0D9QV27 A0A0D9QZZ6 A0A0D9RBZ8 | Uncharacterized protein,VWFA domain-containing protein,Cathepsin D |
| map04975 | Fat digestion and absorption | 0.21780532680881 | 0.7057128651632 | 1 | 10 | 69 | 2848 | Over | A0A0D9S760 | Phospholipid phosphatase 3 |
| map05320 | Autoimmune thyroid disease | 0.21780532680881 | 0.7057128651632 | 1 | 10 | 69 | 2848 | Over | A0A0D9RQI3 | B-cell surface antigen CD40 |
| map05330 | Allograft rejection | 0.21780532680881 | 0.7057128651632 | 1 | 10 | 69 | 2848 | Over | A0A0D9RQI3 | B-cell surface antigen CD40 |
| map04071 | Sphingolipid signaling pathway | 0.226605048446899 | 0.7057128651632 | 3 | 68 | 69 | 2848 | Over | A0A0D9QWC2 A0A0D9RBZ8 A0A0D9RVZ2 | Sphingomyelin phosphodiesterase,Cathepsin D,Ceramidase |
| map04217 | Necroptosis | 0.226605048446899 | 0.7057128651632 | 3 | 68 | 69 | 2848 | Over | A0A0D9QWC2 A0A0D9RWK4 A0A0D9S334 | Sphingomyelin phosphodiesterase,Ubiquitinyl hydrolase 1,Ferritin |
| map05202 | Transcriptional misregulation in cancer | 0.252565028016261 | 0.730870061815483 | 2 | 40 | 69 | 2848 | Over | A0A0D9RQI3 A0A0D9RRB2 | B-cell surface antigen CD40,Plasminogen activator |
| map00531 | Glycosaminoglycan degradation | 0.273500865429385 | 0.730870061815483 | 1 | 13 | 69 | 2848 | Over | A0A0D9RP19 | Hyaluronidase |
| map05020 | Prion diseases | 0.273500865429385 | 0.730870061815483 | 1 | 13 | 69 | 2848 | Over | A0A0D9S2Z2 | Neural cell adhesion molecule 1 |
| map05143 | African trypanosomiasis | 0.273500865429385 | 0.730870061815483 | 1 | 13 | 69 | 2848 | Over | A0A0D9S6M6 | Uncharacterized protein |
| map00100 | Steroid biosynthesis | 0.291182854948035 | 0.730870061815483 | 1 | 14 | 69 | 2848 | Over | A0A0D9QXG6 | Cytochrome P450 family 2 subfamily R member 1 |
| map04745 | Phototransduction - fly | 0.291182854948035 | 0.730870061815483 | 1 | 14 | 69 | 2848 | Over | A0A0D9R294 | Lipase\_3 domain-containing protein |
| map01521 | EGFR tyrosine kinase inhibitor resistance | 0.297975217523258 | 0.730870061815483 | 2 | 45 | 69 | 2848 | Over | A0A0D9RXU0 A0A0D9S0W1 | Growth arrest specific 6,Receptor protein-tyrosine kinase |
| map04510 | Focal adhesion | 0.298527178309471 | 0.730870061815483 | 4 | 98 | 69 | 2848 | Over | A0A0D9R486 A0A0D9R9X4 A0A0D9RY11 A0A0D9RY12 | Collagen type IV alpha 3 chain,Fibronectin,Collagen type IV alpha 2 chain,Collagen IV NC1 domain-containing protein |
| map00760 | Nicotinate and nicotinamide metabolism | 0.308440576545984 | 0.730870061815483 | 1 | 15 | 69 | 2848 | Over | A0A0D9RXR7 | Ecto-5-nucleotidase |
| map04964 | Proximal tubule bicarbonate reclamation | 0.308440576545984 | 0.730870061815483 | 1 | 15 | 69 | 2848 | Over | A0A0D9RQJ5 | Aquaporin-1 |
| map00590 | Arachidonic acid metabolism | 0.325284064092163 | 0.738665895542621 | 1 | 16 | 69 | 2848 | Over | A0A0D9RRE1 | Glutathione peroxidase |
| map04640 | Hematopoietic cell lineage | 0.325284064092163 | 0.738665895542621 | 1 | 16 | 69 | 2848 | Over | A0A0D9S6I0 | Macrophage colony-stimulating factor 1 |
| map04922 | Glucagon signaling pathway | 0.334057486749799 | 0.743107470525062 | 2 | 49 | 69 | 2848 | Over | A0A0D9R6H1 A0A0D9S336 | Glycogen [starch] synthase,Glycogen [starch] synthase |
| map00790 | Folate biosynthesis | 0.341723117615341 | 0.744956396401444 | 1 | 17 | 69 | 2848 | Over | A0A0D9S8B5 | Alkaline phosphatase |
| map05322 | Systemic lupus erythematosus | 0.35776730867311 | 0.753807121564877 | 1 | 18 | 69 | 2848 | Over | A0A0D9RQI3 | B-cell surface antigen CD40 |
| map04722 | Neurotrophin signaling pathway | 0.360794763096624 | 0.753807121564877 | 2 | 52 | 69 | 2848 | Over | A0A0D9RJT1 A0A0D9S6J4 | MAGE domain-containing protein,Sortilin 1 |
| map00565 | Ether lipid metabolism | 0.388708278633614 | 0.770349134019344 | 1 | 20 | 69 | 2848 | Over | A0A0D9S760 | Phospholipid phosphatase 3 |
| map04979 | Cholesterol metabolism | 0.388708278633614 | 0.770349134019344 | 1 | 20 | 69 | 2848 | Over | A0A0D9S6J4 | Sortilin 1 |
| map04924 | Renin secretion | 0.418179173471822 | 0.813955891221939 | 1 | 22 | 69 | 2848 | Over | A0A0D9RQJ5 | Aquaporin-1 |
| map04910 | Insulin signaling pathway | 0.436349171382439 | 0.834422099661155 | 3 | 78 | 69 | 2848 | Over | A0A0D9R6H1 A0A0D9RU34 A0A0D9S336 | Glycogen [starch] synthase,CBM21 domain-containing protein,Glycogen [starch] synthase |
| map00330 | Arginine and proline metabolism | 0.459778899331573 | 0.836156969901148 | 1 | 25 | 69 | 2848 | Over | A0A0D9R8E6 | Procollagen-proline 4-dioxygenase |
| map05416 | Viral myocarditis | 0.459778899331573 | 0.836156969901148 | 1 | 25 | 69 | 2848 | Over | A0A0D9RQI3 | B-cell surface antigen CD40 |
| map04115 | p53 signaling pathway | 0.472983028253331 | 0.836156969901148 | 1 | 26 | 69 | 2848 | Over | A0A0D9QZU3 | Tetraspanin |
| map05323 | Rheumatoid arthritis | 0.472983028253331 | 0.836156969901148 | 1 | 26 | 69 | 2848 | Over | A0A0D9S6I0 | Macrophage colony-stimulating factor 1 |
| map04918 | Thyroid hormone synthesis | 0.498444327473893 | 0.848912995228973 | 1 | 28 | 69 | 2848 | Over | A0A0D9RRE1 | Glutathione peroxidase |
| map04976 | Bile secretion | 0.498444327473893 | 0.848912995228973 | 1 | 28 | 69 | 2848 | Over | A0A0D9RQJ5 | Aquaporin-1 |
| map04216 | Ferroptosis | 0.51071643435485 | 0.856432174533518 | 1 | 29 | 69 | 2848 | Over | A0A0D9S334 | Ferritin |
| map00561 | Glycerolipid metabolism | 0.534379602043177 | 0.879762921276101 | 1 | 31 | 69 | 2848 | Over | A0A0D9S760 | Phospholipid phosphatase 3 |
| map00480 | Glutathione metabolism | 0.545784574517093 | 0.879762921276101 | 1 | 32 | 69 | 2848 | Over | A0A0D9RRE1 | Glutathione peroxidase |
| map04620 | Toll-like receptor signaling pathway | 0.556914142826156 | 0.879762921276101 | 1 | 33 | 69 | 2848 | Over | A0A0D9RQI3 | B-cell surface antigen CD40 |
| map05110 | Vibrio cholerae infection | 0.556914142826156 | 0.879762921276101 | 1 | 33 | 69 | 2848 | Over | A0A0D9RRA4 | Uncharacterized protein |
| map04137 | Mitophagy - animal | 0.578373132860275 | 0.887924950447465 | 1 | 35 | 69 | 2848 | Over | A0A0D9RQT0 | Uncharacterized protein |
| map04925 | Aldosterone synthesis and secretion | 0.578373132860275 | 0.887924950447465 | 1 | 35 | 69 | 2848 | Over | A0A0D9R294 | Lipase\_3 domain-containing protein |
| map00310 | Lysine degradation | 0.588715206743191 | 0.891249410208441 | 1 | 36 | 69 | 2848 | Over | A0A0D9R3X6 | Histone-lysine N-methyltransferase EZH2 |
| map05120 | Epithelial cell signaling in Helicobacter pylori infection | 0.598807187801057 | 0.894109362607057 | 1 | 37 | 69 | 2848 | Over | A0A0D9R3N7 | GIT ArfGAP 1 |
| map05132 | Salmonella infection | 0.6086550369799 | 0.896532419335257 | 1 | 38 | 69 | 2848 | Over | A0A0D9RQT0 | Uncharacterized protein |
| map05418 | Fluid shear stress and atherosclerosis | 0.650650338494414 | 0.945611825278548 | 2 | 58 | 69 | 2848 | Over | A0A0D9RRB2 A0A0D9S6M6 | Plasminogen activator,Uncharacterized protein |
| map04152 | AMPK signaling pathway | 0.667602763079528 | 0.957482910206165 | 2 | 64 | 69 | 2848 | Over | A0A0D9R6H1 A0A0D9S336 | Glycogen [starch] synthase,Glycogen [starch] synthase |
| map04140 | Autophagy - animal | 0.696607941213517 | 0.982939164867871 | 2 | 73 | 69 | 2848 | Over | A0A0D9RBZ8 A0A0D9RQT0 | Cathepsin D,Uncharacterized protein |
| map04014 | Ras signaling pathway | 0.703387659263248 | 0.982939164867871 | 2 | 75 | 69 | 2848 | Over | A0A0D9R3Z2 A0A0D9S6I0 | Lipase domain-containing protein,Macrophage colony-stimulating factor 1 |
| map04810 | Regulation of actin cytoskeleton | 0.732616829891691 | 0.998190430727429 | 3 | 99 | 69 | 2848 | Over | A0A0D9QZZ6 A0A0D9R3N7 A0A0D9R9X4 | VWFA domain-containing protein,GIT ArfGAP 1,Fibronectin |
| map05165 | Human papillomavirus infection | 0.77567900275068 | 1 | 4 | 142 | 69 | 2848 | Over | A0A0D9R486 A0A0D9R9X4 A0A0D9RY11 A0A0D9RY12 | Collagen type IV alpha 3 chain,Fibronectin,Collagen type IV alpha 2 chain,Collagen IV NC1 domain-containing protein |
| map04145 | Phagosome | 1 | 1 | 2 | 81 | 69 | 2848 | Over | A0A0D9QV27 A0A0D9RQT0 | Uncharacterized protein,Uncharacterized protein |
| map04380 | Osteoclast differentiation | 1 | 1 | 1 | 40 | 69 | 2848 | Over | A0A0D9S6I0 | Macrophage colony-stimulating factor 1 |
| map04520 | Adherens junction | 1 | 1 | 1 | 41 | 69 | 2848 | Over | A0A0D9RYA9 | Protein-tyrosine-phosphatase |
| map05215 | Prostate cancer | 1 | 1 | 1 | 40 | 69 | 2848 | Over | A0A0D9RRB2 | Plasminogen activator |
| map05230 | Central carbon metabolism in cancer | 1 | 1 | 1 | 40 | 69 | 2848 | Over | A0A0D9RQF9 | Protein-serine/threonine kinase |
